# Interclonal differences in incipient limiting level (ILL) in *Daphnia magna*

**DOI:** 10.1093/plankt/fbag022

**Published:** 2026-04-23

**Authors:** Anna Bednarska, Joanna Pijanowska, Barbara Pietrzak

**Affiliations:** Department of Hydrobiology, Institute of Ecology, Faculty of Biology, University of Warsaw, at Biological and Chemical Research Centre, Żwirki i Wigury 101, 02-089 Warszawa, Poland; Department of Hydrobiology, Institute of Ecology, Faculty of Biology, University of Warsaw, at Biological and Chemical Research Centre, Żwirki i Wigury 101, 02-089 Warszawa, Poland; Department of Hydrobiology, Institute of Ecology, Faculty of Biology, University of Warsaw, at Biological and Chemical Research Centre, Żwirki i Wigury 101, 02-089 Warszawa, Poland

**Keywords:** Daphnids, growth rate, intrinsic rate of increase, fitness, food quantity, incipient limiting level

## Abstract

Seasonal variation in phytoplankton biomass imposes strong selection pressures on planktonic herbivores such as *Daphnia*, with both low and high food availability shaping life-history strategies. While the threshold food level (TFL) has been widely studied, the incipient limiting level (ILL)—the food concentration above which growth or reproduction no longer increases—remains poorly characterized at the intraspecific level. This study aimed to quantify clonal variation in ILL among four genetically distinct *Daphnia magna* clones originating from pond and lake habitats. Individuals were reared along a gradient of algal concentrations under standardized laboratory conditions, and somatic growth rate and intrinsic rate of increase were calculated. Results showed substantial among-clone variation in ILL for somatic growth rate, spanning ~ 0.7–1 mg C_org_L^−1^. Similar patterns were observed for population growth rates, with one clone requiring significantly higher food levels (~1.3 mg C_org_L^−1^) to achieve maximum fitness. These findings challenge the general assumption of a universal ILL and suggest that its clonal variation is generated by local adaptation to food regimes. Incorporating clone-specific ILLs into ecological models may improve predictions of population dynamics and community composition under fluctuating trophic conditions.

## INTRODUCTION

In lakes of temperate zone, feeding conditions for planktonic herbivores change seasonally, with maxima of phytoplankton biomass in spring and summer separated by a clear water phase, during which food for planktonic animals is extremely scarce (PEG model; Sommer *et al.*, 1986; Sommer *et al.*, 2012). Under such cyclical resource supply, the ability to cope with and thrive under both near-starvation and favorable food conditions can be crucial for the fitness of the animal. On the lower end, it has been shown that threshold food level (TFL, concentration of food at which growth rate equals zero) is essential for fitness and determines the competitive ability in different *Daphnia* species (Gliwicz, 1990, 2003; Gliwicz and Lampert, 1990; Achenbach and Lampert, 1997; Tessier *et al.*, 2000) and between clones within a species (Przytulska *et al.*, 2015; Maruoka and Urabe, 2020).

Similarly, the ability to exploit food at high concentrations should be subject to selection pressure, as physiological adaptations that enable increased production under phytoplankton abundance provide a fitness advantage over competitors. The ability to fully exploit high spring food abundance should translate into a demographic response, increasing the chances for monopolizing the resources. However, this efficient use of resources leads to overcrowding and overexploitation of phytoplankton biomass resulting in the clear water phase (PEG model; Sommer *et al.*, 1986; Sommer *et al.*, 2012). To survive approaching periods of food scarcity, organisms may adopt one of three main strategies: *r*-selection, *K*-selection, or quiescence. The effectiveness of each strategy depends on how efficiently resources are utilized. First, higher fecundity, and hence reproductive rate, increases chances for individual and clonal survival through food shortages. Second, *Daphnia* are able to react to overcrowding cues and prepare offspring for hunger conditions with additional allocation of resources to the eggs (Mikulski and Pijanowska, 2023). Third is producing diapausing eggs to bide the time of food scarcity (Vanoverbeke and De Meester, 2009). Genotypes most efficiently profiting from food resources at their highest abundance can invest more in these “preparatory” strategies.

The food quantity at which maximal values of fitness parameters are attained is defined as incipient limiting level (ILL) (Lampert, 1978). The ILL was initially defined as the lowest food density that enables the maximum ingestion rate (McMahon and Rigler, 1963), however the meaning of this term has broadened, often becoming synonymous with the saturation threshold for any fitness trait (e.g. Lampert, 1978; Müller-Navarra, 1995; Ślusarczyk, 2001; Martin-Creuzburg *et al.*, 2005). Above ILL, fitness, whether measured by somatic growth rate, carbon content, egg production or population growth rate, no longer increases. Ecologically, ILL sets the boundary between food-saturated and food-limited conditions for grazers and therefore should strongly influence herbivore population dynamics, herbivore–algae interactions, competition among consumers, and the strength of top–down control in pelagic food webs. The ability to effectively utilize food resources at and above ILL, together with differences in fitness values attained at this level, may therefore constitute targets for selection. Under predation, these traits can determine the capacity for demographic compensation and, consequently, the relative frequencies of genotypes.

There are several physiological mechanisms that may facilitate the efficacy of utilizing abundant resources: filtration rate, ingestion rate, efficiency of digestion, assimilation and respiratory rates, among others. These parameters do differ between *Daphnia* species (Burns, 1969; Lynch *et al.*, 1986;) and clones (Glazier and Calow, 1992). Among these, both assimilation and respiratory rate increase with increasing food concentration until a plateau is reached (Bohrer and Lampert, 1988). An organism for which the balance between assimilation and respiration plateaus at highest food concentration should have the highest ILL. Yet, despite well-documented differences among clones and species in assimilation and respiratory rates, the same food concentration of food (expressed in C_org_L^−1^ units) is commonly used as at the “control” across *Daphnia* studies. This practice can be traced back to the seminal paper by Lampert (1978), in which the number of egg productions per female “saturated”—at approximately 0.7 mg C_org_L^−1^ when *D. pulex* were fed *Scenedesmus acutus*. To provide a safety margin, a concentration of 1 mg C_org_L^−1^ has since been widely adopted as a standard control level, often referred to as *ad libitum*, implicitly assumed to be above the ILL for *Daphnia*, regardless of species (Repka, 1997; Becker and Boersma, 2003; Rellstab and Spaak, 2007; De Senerpont Domis *et al.*, 2013; Lind and Jeyasingh, 2018; Dinh *et al.*, 2018; Yousey *et al.*, 2018; Zhang *et al.*, 2021; among countless others). Lampert (1978), already noted that the ILL may shift depending on *Daphnia* species, predation regime or other environmental factors influencing mortality. Nevertheless, the widespread use of a single reference concentration has considerable practical value: as a shared benchmark it facilitates comparability across studies and experimental systems.

Despite the fact that the ILL of food—defined here as the algal concentration allowing maximum somatic and population growth—can be a key determinant of both individual performance and population-level fitness, comparative data on its values across different genotypes remain limited, particularly under standardized conditions. The aim of this study was to detect the expected genetic variability in ILL. We compared the performance of four different clones of *Daphnia magna* along a gradient of food quantity, to eventually determine clone-specific ILL (and its plateau elevation), hypothesizing that, like threshold food concentration, also ILL differs between clones.

## MATERIALS AND METHODS

The experiment was performed with individuals of *Daphna magna* belonging to four clonal lineages. Two clones: B2 and B3 originated from Oud Heverlee Pond (central Belgium, 50°50′22′′N, 4°39′18′′E) and 2 clones: D2 and D4 from Binnesee (north-eastern Germany; 54°19′29″N 10°37′39″E). The goal of the experiment was to test whether 1 mg C_org_L^−1^ is indeed a universal, non-limiting food concentration for individuals of different clones. We treated clones as independent experimental units, and the inclusion of clones originating from different localities was intended to increase the robustness of the analysis. Each clonal culture was established from a single female hatched from ephippium. Before the experiment, *Daphnia* were cultured for at least four generations in a temperature-controlled water bath (20°C ± 0.5°C), summer photoperiod (16 L:8D), and fed daily with a suspension of *Acutodesmus obliquus* (SAG 276-3a; also known as *Scenedesmus obliquus* or *S. acutus f. alternan*s, *Tetradesmus obliquus*) with a density corresponding to 1 mg C_org_L^−1^. The culture medium was exchanged every second day. Second clutch offspring were taken for further culture.

The media used in pre-experimental culture and during the experiment were prepared using aged water from Janówek pond (located near Warsaw). The water was stored in a large, aerated tank for two weeks prior to use. Before preparing the culturing and experimental media, the water was re-filtered through a 0.2-μm pore-sized capsule filter (Sartobran® P) and then enriched with a suspension of green algae particles of the required concentration.

Synchronized (born within 14 hours) subsamples of neonates (six replications of 5 neonates for each clone) of each clone were dried and then weighed (Orion Cahn C-35 Ultra-Microbalance, Thermo Electron Corporation, USA) to assess the initial body mass, and the rest of neonates were randomly distributed to one of twelve experimental treatments of different green algae concentration. The initial concentrations of *A. obliquus* were as follows: 0.0125; 0.025; 0.05; 0.1; 0.15; 0.25; 0.35; 0.5; 1.0; 1.5; 2.5 and 4.5 mg C_org_L^−1^. Thus, they ranged from the concentration close to the threshold food concentration for *D. magna* growth (Gliwicz, 1990) to concentration well above the concentration at which *Daphnia* should still be food limited (Lampert, 1978). In this experimental design, food the concentration decreased over time, however, the assumption was that it declined fairly evenly across different clones, thus the interclonal comparisons are justified. Moreover, the volume in which the *Daphnia* were kept was calculated so that even adult *Daphnia* were unable to filter the entire volume of medium within 24 hours. Individuals were kept separately in 100 mL vessels. The abiotic conditions were the same as in pre-experimental cultures. There were ten replications of each food concentration for each clone, adding up to 480 individuals (4 clones×12 food concentrations×10 replications) in total. The animals were inspected every day at the same time (around 10 a.m.), during the exchange of the media. Females were cultured in given conditions until first eggs were deposited into the brood chamber. Females with eggs in the brood chamber were marked for further processing. If a female deposited eggs after the inspection, she was considered mature the next day. Age at first reproduction and number of eggs (*N_eggs_*), i.e. the life history parameters later used for calculating population growth rate were monitored. The number of eggs was counted intravitally (the three-dimensional image from under the binocular microscope allows for reliable assessment of the number of eggs), then the females were photographed (binocular microscope equipped with digital camera) and the captured images were analysed using the NIS-Elements software (Nikon, Tokyo, Japan). To calculate the individual growth rate (*g_i_*), the egg-bearing females were dried (24 hours, 60°C) and weighed. The initial and final dry masses were converted into growth rates per day using the formula *g_i_* = (ln[*M_t_*]—ln[*M_0_*])/*t* (Lampert and Trubetskova, 1996), where *M_0_* and *M_t_* are the dry masses of the animals at day zero and at the end of the experiment, respectively, and *t* is the duration of the experiment (here equal to age at first reproduction, in days, individually for each female). Intrinsic rate of increase (*r*, day^−1^) was calculated using the Euler–Lotka equation (1 = Σ e^-*rx*^  *l_x_ m_x_*), where *x* is age in days at first reproductive event, *l_x_* is the probability of surviving to age *x*, and *m_x_* is the number of offspring produced at age *x*. This equation was solved numerically for *r* using the uniroot() function in R.

The intrinsic rate of increase estimated here reflects early-life population growth potential, as it integrates survival to, and fecundity at, the first reproductive event, rather than complete lifetime reproduction.

Individual somatic growth rate (*g_i_*) and population growth rate (*r*) were analysed as functions of food concentration using a quadratic plateau model. The model includes three parameters: an intercept (*b₀*), a slope describing the initial increase in performance with food availability (*b₁*), and a break-point (*c_x_*), interpreted as the ILL. Model fitting was performed separately for each clone using nonlinear least-squares estimation implemented in the nls() function in R; when convergence was not achieved, the Levenberg–Marquardt algorithm (nlsLM()) was used as a fallback. The quadratic term was derived from the fitted parameters, yielding a concave-down saturation curve approaching a plateau value, interpreted as the maximal performance under non-limiting food conditions. The plateau height was calculated as the predicted response at the ILL. In addition to ILL, the quadratic plateau model also allowed estimation of the TFL, defined as the x-intercept of the fitted function (i.e. the food concentration at which predicted performance equals zero).

Uncertainty in parameter estimates was quantified using nonparametric bootstrap resampling (1 000 iterations). For somatic growth, bootstrap resampling was performed at the level of individual observations within each clone × food concentration combination. For population growth rate (*r*), which was derived from life-history traits, bootstrap resampling was performed at the individual level by resampling fecundity and age at first reproduction prior to recalculating *r* via the Euler–Lotka equation. Bootstrap distributions were used to derive 95% confidence intervals for model coefficients and biologically interpretable parameters, including ILL and plateau height. Quadratic plateau function with three parameters—intercept, slope and break-point—was fitted to individual growth rate (*g_i_*) and population growth rate (*r*) data using a self-starter function SSquadp3xs() from the nlraa package (Miguez, 2023). The nonlinear least-squares estimates of the parameters of the quadratic plateau model were determined using nls() function. 95% confidence intervals (CIs) for break-points coordinates (*x*: ILL, *y*: plateau elevation) were estimated using the bootstrap method with 1 000 resamples. To assess the robustness of our estimates to model structure, we additionally fitted a simpler piecewise linear (“hockey-stick”) model with an abrupt threshold. The results are shown in Supplementary Material (Fig. S1–S2, Table S3).

The analyses were performed and the results plotted using R language and environment (R Core Team 2025).

## RESULTS

**Fig. 1:**
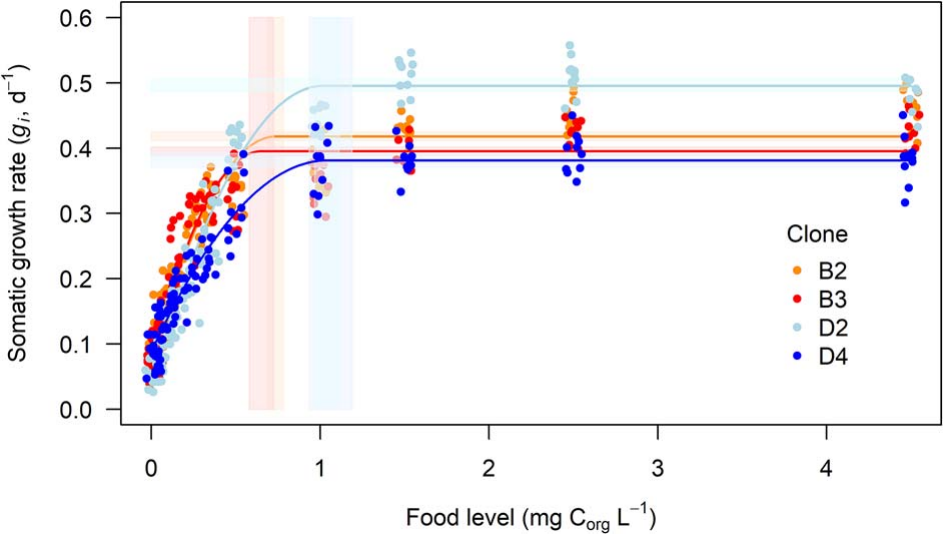
Somatic growth rate (*g_i_*) in females of four *D. magna* clones along the gradient of 12 *A. obliquus* concentrations. Points—individual data. Lines—fitted quadratic plateau functions. Shaded bands—95% confidence intervals for the estimated ILL (vertical) and plateau (horizontal) values.

The individual growth rate plateaued at lower food concentrations (i.e. ILL for somatic growth was lower) in *Daphnia* clones originating from the pond (ca. 0.7 mg C_org_L^−1^) than those from the lake (ca. 1 mg C_org_L^−1^): B3 = B2 < D2 = D4 (based on estimate confidence intervals; Table 1, Fig. 1). The maximum individual growth rate also differed between clones: D4 = B3 < B2 < D2 (Table 1, Fig. 1).

**Table I:** *Plateau breakpoint (ILL) and elevation estimates and 95% confidence intervals (CI) for individual growth rate (*g_i_*) and population growth rate (*r*) for the four studied* D. magna *clones*

clone	ILL (mg C L ^−1^)		Plateau elevation	
	Estimate	95% CI	Estimate	95% CI
	***Individual growth rate (g***_***i***_ ***)***			
B2	0.739	[0.694, 0.783]	0.418	[0.412, 0.424]
B3	0.651	[0.574, 0.723]	0.395	[0.389, 0.401]
D2	1.02	[0.97, 1.12]	0.496	[0.487, 0.506]
D4	1.05	[0.94, 1.19]	0.381	[0.370, 0.393]
	***Population growth rate (r)***			
B2	0.613	[0.576, 0.668]	0.390	[0.387, 0.398]
B3	0.659	[0.622, 0.732]	0.362	[0.357, 0.372]
D2	0.708	[0.658, 0.740]	0.417	[0.409, 0.427]
D4	1.287	[0.945, 1.678]	0.327	[0.299, 0.334]

**Fig. 2:**
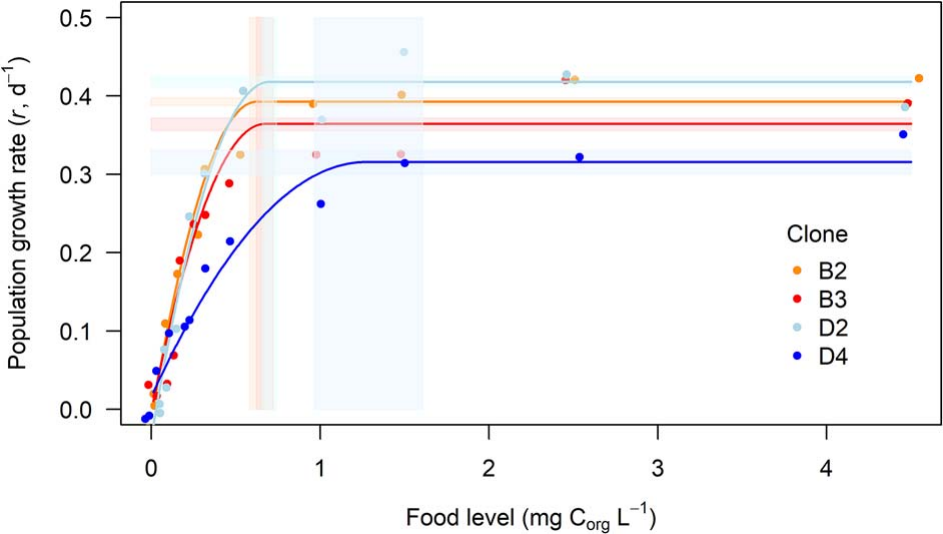
Early-life intrinsic rate of increase (*r*) in cohorts of four *D. magna* clones along the gradient of 12 *A. obliquus* concentrations. Points—individual data. Lines—fitted quadratic plateau functions. Shaded bands—95% confidence intervals for the estimated ILL (vertical) and plateau (horizontal) values.

The population growth rate plateaued at higher food concentration (i.e. ILL for population growth was higher) in D4 (ca. 1.3 mg C_org_L^−1^) than in other clones (ca. 0.6–0.7 mg C_org_L^−1^): B2 = B3 = D2 < D4 (Table 1, Fig. 2). The maximum population growth rate ranged from 0.33 to 0.42 and ordered the clones similarly to individual growth: D4 < B3 < B2 < D2 (Table 1, Fig. 2).

Detailed parameter estimates of the fitted quadratic plateau models, including coefficients, confidence intervals and measures of model fit are provided in Supplementary Tables S1–S2. For completeness, we also report TFLs (*x*-intercepts of the fitted functions) and survival to first reproduction (*l_x_*). Both are presented in detail in the Supplementary Materials (Tables S1–S2, S4). Survival to first reproduction (*l_x_*) was consistently high across food concentrations and did not show a systematic decline at low food levels in any clone (Table S4), indicating that variation in ILL and TFL estimates was not driven by food-dependent mortality.

## DISCUSSION

In life history, the ILL of food above which the production does not increase was identified as 0.7 C_org_L^−1^ for *D. pulex* (Lampert, 1978), and a concentration of 1 mg C_org_L^−1^ has been used as a standard in laboratory studies in which *Daphnia* were considered to be fed ad libitum. Yet, as we show here, *Daphnia* clones differ in the ILL (Figs. 1 and 2; Tables S1 and S2), as they differ in the threshold food concentration. Clonal variation in non-linear plastic responses was visible for both, individual somatic growth rate (*g_i_*) and early-life intrinsic rate of increase (*r*), and the nature and magnitude of variation along the food gradient was trait-specific, as it was recently demonstrated for *Daphnia* showing multi-trait flexible responses across the food gradient (Plaistow *et al.*, 2022). For most of the clones studied here, 1 mg C_org_L^−1^ indeed served as ad libitum, this was however not true for all.

Interestingly, as in Lampert’s *D. pulex*’s egg production (1977) and carbon content (1976), ILL for early-life intrinsic rate of increase measured here roughly fell around 0.7 mg C_org_L^−1^ in most *D. magna* clones studied (B2, B3, D2), and for somatic individual growth rate in half of them (B2, B3). Yet, in the small sample of four clones there was one (25%) which saturated at higher food concentration in both parameters. By no means 0.7 mg C_org_L^−1^ provides *ad libitum* conditions for growth of females in this clonal lineage, although 1 mg C_org_L^−1^ already does. However, it can be expected that in a larger clonal sample, clones with even higher ILL will be found, where 1 mg C_org_L^−1^ would not suffice to provide ad libitum conditions. The one outlier (D4) seems to be a low-food specialist (or “cyanobacteria specialists” as shown in our previous studies; Bednarska *et al.*, 2014; Bednarska, 2022), with a competitive advantage in times of scarcity (see the lowest threshold food concentration, Table S1), but probably being outcompeted at higher food concentrations, presumably due to slower maximum ingestion/filtration/digestion rates or higher respiratory rate. This hypothesis would have to be tested in an experiment where individuals of different clones could compete directly. A low growth rate at high food concentrations does not necessarily indicate competitive inferiority, as competitive outcomes in *Daphnia* can be strongly influenced by environmental factors such as food quality, nutrient stoichiometry, or genotype–environment interactions (Weider *et al.*, 2005; Feniova *et al.*, 2025).

Even where ILL was similar in different clones, the plateau for individual and population growth rates was reached at different values. This points to possible physiological limitations that preclude individuals of given clones from attaining growth above that plateau. This is, in consequence, crucial for clonal composition of planktonic community, as some clones attain higher growth rates and relative abundances, thus implying high food concentration to be a strong determinant of clonal selection. In situations of high external mortality, the differences in maximum growth rates at and above ILL displayed by individuals of different clones can be critical. Under intense fish predation, the ability to exploit food abundance effectively to achieve a higher growth rate than conspecifics can facilitate numerical response and demographic compensation. Hence, our results strongly suggest that both threshold food concentration and ILL determine clonal success under seasonally changing food conditions.

Finally, it seems that clones differ in ILL values, possibly reflecting local adaptation to food regimes and associated trade-offs in energy allocation. This issue is worth further investigation.

Body size, not studied here, is another factor influencing *Daphnia* efficiency in food utilization (e.g. Brooks and Dodson, 1965; Urabe and Watanabe, 1991). Different species of *Daphnia* exhibit varying ILLs depending on their size (Lampert, 1987), and metabolic balance between assimilation and respiration. Smaller species, such as *Daphnia* belonging to *D. longispina* complex, should have lower ILLs due to lower filtration and assimilation rates (Burns, 1969; Lynch *et al.*, 1986). Thus, it can be expected that the food level of 1 mg C_org_L^−1^ will represent an overfeeding condition for smaller species. This can be inferred from the results presented in Pereira and Gonçalves (2008), who traced egg production and somatic growth in a gradient of food concentrations in three *D. longispina* and one *D. magna* clones. In their studied food concentration range, the fecundity of one of *D. longispina* clones was saturated at relatively low food concentration while this parameter did not reach a plateau in the *D. magna* clone (Pereira and Gonçalves, 2008).

The ILL is a key ecological parameter that links resource availability with individual fitness and demographic performance of *Daphnia*. Its variation across clones and species underpins fundamental processes such as niche differentiation, competition and adaptation to changing environments. The ecological relevance of ILL lies in its ability to predict population structure and its shifts under changing trophic conditions. In mesotrophic and eutrophic lakes, co-occurrence of multiple *Daphnia* genotypes may be mediated by spatial or temporal segregation along food gradients defined by clone- and species-specific ILLs. Incorporating clonal ILL variability into dynamic energy budget models and individual-based simulations may improve predictions of population responses to food pulses, eutrophication, or climate-driven changes in phytoplankton phenology. Also, it has been shown that the co-limitation with other environmental factors can occur (Sperfeld *et al.*, 2012). Sperfeld and Wacker (2011) showed that temperature or even the availability of a single essential lipid (cholesterol) can affect the growth saturation thresholds. Additionally, studies using a concentration of *Acutodesmus obliquus* (formerly known as *S. obliquus*) greater than 1 C_org_L^−1^ as a control (e.g. Freese and Martin-Creuzburg, 2013; Bednarska *et al.*, 2014) found that adding an alternative food source, such as heterotrophic bacteria or non-toxic, edible cyanobacteria, further improved *D. magna* growth. Future work should focus on quantifying ILL plasticity under varying food quality, temperature and predation pressure to integrate physiological thresholds into broader ecological and evolutionary frameworks.

## CONCLUSIONS

The ILL—defined as the algal food concentration above which fitness parameters no longer increase—has been widely accepted in experimental aquatic ecology. However, our study challenges this assumption by demonstrating substantial clonal variability in ILL within *D. magna*. Our results reveal that clones differ significantly in the food level at which growth saturates. These findings suggest local adaptation to contrasting trophic regimes and call for a reevaluation of the commonly used standard “*ad libitum*” feeding levels in laboratory studies.

## Supplementary Material

fbag022_Supplemental_Files

## Data Availability

The raw data that support the findings of this study is openly available in RepOD, https://doi.org/10.18150/3ILNJK.

## Notes

Corresponding editor: Lars-Anders Hansson
