# Supplementary material for: Interclonal differences in incipient limiting level (ILL) in Daphnia magna
Source: J Plankton Res. 2026 Apr 23;48(3):fbag022. doi: 10.1093/plankt/fbag022 (PMC13104730; doi:10.1093/plankt/fbag022)
Supplement: fbag022_Supplemental_Files [file fbag022_supplemental_files.zip › SupFig_captions_fbag022.docx]

Fig. S1 Individual somatic growth rate (*g_i_*) in females of four *D. magna* clones along the gradient of 12 *A. obliquus* concentrations. Points - individual data. Solid Lines - fitted quadratic plateau (QP) functions; dashed lines - fitted piecewise linear (“hockey-stick”, HS) functions.

Fig. S2 Early-life intrinsic rate of increase (*r*) in cohorts of four *D. magna* clones along the gradient of 12 *A. obliquus* concentrations. Points - individual data. Solid Lines - fitted quadratic plateau (QP) functions; dashed lines - fitted piecewise linear (“hockey-stick”, HS) functions.
