# Supplementary material for: Interclonal differences in incipient limiting level (ILL) in Daphnia magna
Source: J Plankton Res. 2026 Apr 23;48(3):fbag022. doi: 10.1093/plankt/fbag022 (PMC13104730; doi:10.1093/plankt/fbag022)
Supplement: fbag022_Supplemental_Files [file fbag022_supplemental_files.zip › Tab._S1_Growth_QP_fbag022.docx]

**Supplementary material**

Table S1. Parameter estimates of the quadratic plateau model fitted to individual somatic growth rate (*g_i_*). Rows represent model parameters and goodness-of-fit metrics; columns represent clones. Estimates are shown with standard errors (SE), 95% confidence intervals (CI), and model fit statistics.

| **Parameter** |  | **B2** | **B3** | **D2** | **D4** |
| --- | --- | --- | --- | --- | --- |
| Sample size (n) |  | 120 | 120 | 114 | 115 |
| **Intercept (b0)** | **Estimate** | **7.8381** | **7.5986** | **3.8089** | **8.7983** |
|  | CI low | 6.5691 | 6.1904 | 2.6296 | 7.6770 |
|  | CI high | 9.0865 | 8.9496 | 5.0350 | 9.9074 |
| **Slope (b1)** | **Estimate** | **91.8895** | **98.1049** | **89.6876** | **56.0453** |
|  | CI low | 82.0613 | 85.8284 | 81.1671 | 49.3334 |
|  | CI high | 102.9063 | 113.3914 | 97.4004 | 63.2486 |
| **Curvature (b2)** | **Estimate** | **-62.1495** | **-75.3419** | **-43.9557** | **-26.7602** |
|  | CI low | -70.8246 | -94.5704 | -48.6488 | -34.1660 |
|  | CI high | -55.4102 | -60.6904 | -37.0368 | -20.8611 |
| **ILL (mg C L⁻¹) *** | **Estimate** | **0.7393** | **0.6511** | **1.0202** | **1.0472** |
|  | CI low | 0.6678 | 0.5704 | 0.9497 | 0.9422 |
|  | CI high | 0.8162 | 0.7331 | 1.1168 | 1.1734 |
| **Plateau (d⁻¹) **** | **Estimate** | **0.4180** | **0.3953** | **0.4956** | **0.3814** |
|  | CI low | 0.4118 | 0.3893 | 0.4864 | 0.3717 |
|  | CI high | 0.4241 | 0.4009 | 0.5063 | 0.3918 |
| **Threshold (mg C L⁻¹) ***** | **Estimate** | **-0.0809** | **-0.0734** | **-0.0416** | **-0.1467** |
|  | CI low | -0.0906 | -0.0870 | -0.0517 | -0.1793 |
|  | CI high | -0.0722 | -0.0595 | -0.0355 | -0.1230 |
|  |  |  |  |  |  |
| R² |  | 0.9444 | 0.9350 | 0.9735 | 0.9381 |
| AIC |  | 623.1553 | 627.3316 | 572.5232 | 581.1518 |

* *breakpoint (cx); ** value at ILL; *** x-intercept*

*The incipient limiting level (ILL) was defined as the breakpoint on the food concentration axis (cx), the plateau corresponds to the value of the fitted function at the breakpoint, and the threshold food concentration to the x-intercept of the fitted function (i.e. the food level at which the predicted growth rate equals zero).*
