# Supplementary material for: Interclonal differences in incipient limiting level (ILL) in Daphnia magna
Source: J Plankton Res. 2026 Apr 23;48(3):fbag022. doi: 10.1093/plankt/fbag022 (PMC13104730; doi:10.1093/plankt/fbag022)
Supplement: fbag022_Supplemental_Files [file fbag022_supplemental_files.zip › Tab._S2_r_QP_fbag022.docx]

**Supplementary material**

*Bootstrap confidence intervals could not be reliably estimated for r due to limited number of observations per clone.*

Table S2. Parameter estimates of the quadratic plateau model fitted to early-life intrinsic rate of increase (*r*). Rows represent model parameters and goodness-of-fit metrics; columns represent clones. Estimates are shown with standard errors (SE), 95% confidence intervals (CI), and model fit statistics.

| **Parameter** |  | **B2** | **B3** | **D2** | **D4** |
| --- | --- | --- | --- | --- | --- |
| Sample size (n) |  | 12 | 12 | 12 | 12 |
| **Intercept (b0)** | **Estimate** | **-1.2879** | **-0.3093** | **-3.6690** | **1.5956** |
|  | CI low | -2.3349 | -1.3643 | -4.3581 | 0.0478 |
|  | CI high | -0.4064 | 0.5401 | -3.0218 | 2.8118 |
| **Slope (b1)** | **Estimate** | **129.9128** | **108.9774** | **130.0527** | **47.0264** |
|  | CI low | 120.2006 | 101.3133 | 122.5471 | 36.7209 |
|  | CI high | 140.6337 | 117.5050 | 138.1520 | 61.676 |
| **Curvature (b2)** | **Estimate** | **-104.0391** | **-80.8004** | **-93.0183** | **-18.4423** |
|  | CI low | -120.3143 | -93.9454 | -104.3258 | -31.7717 |
|  | CI high | -90.2198 | -70.1597 | -83.0004 | -11.0933 |
| **ILL (mg C L⁻¹) *** | **Estimate** | **0.6243** | **0.6744** | **0.6991** | **1.2750** |
|  | CI low | 0.5823 | 0.6244 | 0.6618 | 0.9674 |
|  | CI high | 0.6698 | 0.7253 | 0.7396 | 1.6512 |
| **Plateau (d⁻¹) **** | **Estimate** | **0.3927** | **0.3644** | **0.4179** | **0.3157** |
|  | CI low | 0.3876 | 0.3559 | 0.4112 | 0.2986 |
|  | CI high | 0.3976 | 0.3720 | 0.4245 | 0.3315 |
| **Threshold (mg C L⁻¹) ***** | **Estimate** | **0.0100** | **0.0028** | **0.0288** | **-0.0335** |
|  | CI low | 0.0032 | -0.0051 | 0.0245 | -0.0720 |
|  | CI high | 0.0174 | 0.0122 | 0.0334 | -0.0008 |
|  |  |  |  |  |  |
| R² |  | 0.9859 | 0.9628 | 0.9856 | 0.9679 |
| AIC |  | 56.6512 | 66.2877 | 59.7072 | 60.0956 |

* *breakpoint (cx); ** value at ILL; *** x-intercept*
