# Supplementary material for: Interclonal differences in incipient limiting level (ILL) in Daphnia magna
Source: J Plankton Res. 2026 Apr 23;48(3):fbag022. doi: 10.1093/plankt/fbag022 (PMC13104730; doi:10.1093/plankt/fbag022)
Supplement: fbag022_Supplemental_Files [file fbag022_supplemental_files.zip › Tab._S3_QP_and_HS_model_comparison_fbag022.docx]

**Supplementary material**

Table S3. Comparison of quadratic plateau (QP) and piecewise linear (“hockey-stick”, HS) models fitted to individual somatic growth rate (*g_i_*) and early-life intrinsic rate of increase (*r*). For each clone, R², incipient limiting level (ILL; mg C L⁻¹), and plateau values are shown for both models. Detailed parameter estimates and confidence intervals for QP models are provided in Tables S1–S2.

| **Parameter** | **Model** | **B2** | **B3** | **D2** | **D4** |
| --- | --- | --- | --- | --- | --- |
| ***Somatic growth rate*** |  |  |  |  |  |
| R² | QP | 0.9444 | 0.9350 | 0.9735 | 0.9381 |
|  | HS | 0.9213 | 0.9183 | 0.9776 | 0.9357 |
|  |  |  |  |  |  |
| ILL (mg C L⁻¹) * | QP | 0.7393 | 0.6511 | 1.0202 | 1.0472 |
|  | HS | 0.5531 | 0.3969 | 0.6341 | 0.6653 |
|  |  |  |  |  |  |
| Plateau (d⁻¹) ** | QP | 0.4180 | 0.3953 | 0.4956 | 0.3814 |
|  | HS | 0.4215 | 0.3875 | 0.4938 | 0.3812 |
| ***Population growth rate*** |  |  |  |  |  |
| R² | QP | 0.9859 | 0.9628 | 0.9856 | 0.9679 |
|  | HS | 0.9762 | 0.9376 | 0.9876 | 0.9617 |
|  |  |  |  |  |  |
| ILL (mg C L⁻¹) * | QP | 0.6243 | 0.6744 | 0.6991 | 1.2750 |
|  | HS | 0.3997 | 0.5204 | 0.4413 | 0.6831 |
|  |  |  |  |  |  |
| Plateau (d⁻¹) ** | QP | 0.3927 | 0.3644 | 0.4179 | 0.3157 |
|  | HS | 0.3866 | 0.3682 | 0.4107 | 0.3072 |

* *breakpoint (cx); ** value at ILL*
