# Supplementary material for: Interclonal differences in incipient limiting level (ILL) in Daphnia magna
Source: J Plankton Res. 2026 Apr 23;48(3):fbag022. doi: 10.1093/plankt/fbag022 (PMC13104730; doi:10.1093/plankt/fbag022)
Supplement: fbag022_Supplemental_Files [file fbag022_supplemental_files.zip › Tab._S4_Survival_fbag022.docx]

**Supplementary material**

Table S4. Survival to first reproduction (𝑙_𝑥_) across food concentrations and *Daphnia* clones. Values represent the proportion of individuals (out of 10) surviving to first reproduction.

| **Parameter** |  | **B2** | **B3** | **D2** | **D4** |
| --- | --- | --- | --- | --- | --- |
| 0.0125 |  | 1 | 1 | 0.8 | 0.9 |
| 0.025 |  | 1 | 1 | 0.9 | 1 |
| 0.05 |  | 1 | 1 | 1 | 0.9 |
| 0.1 |  | 1 | 1 | 1 | 1 |
| 0.15 |  | 1 | 1 | 1 | 0.9 |
| 0.25 |  | 1 | 1 | 1 | 0.9 |
| 0.35 |  | 1 | 1 | 1 | 1 |
| 0.5 |  | 1 | 1 | 1 | 1 |
| 1 |  | 1 | 1 | 0.9 | 1 |
| 1.5 |  | 1 | 1 | 1 | 0.9 |
| 2.5 |  | 1 | 1 | 1 | 1 |
| 4.5 |  | 1 | 1 | 0.8 | 1 |
