# Supplementary figures and images for: Interclonal differences in incipient limiting level (ILL) in Daphnia magna
Source: J Plankton Res. 2026 Apr 23;48(3):fbag022. doi: 10.1093/plankt/fbag022 (PMC13104730; doi:10.1093/plankt/fbag022)

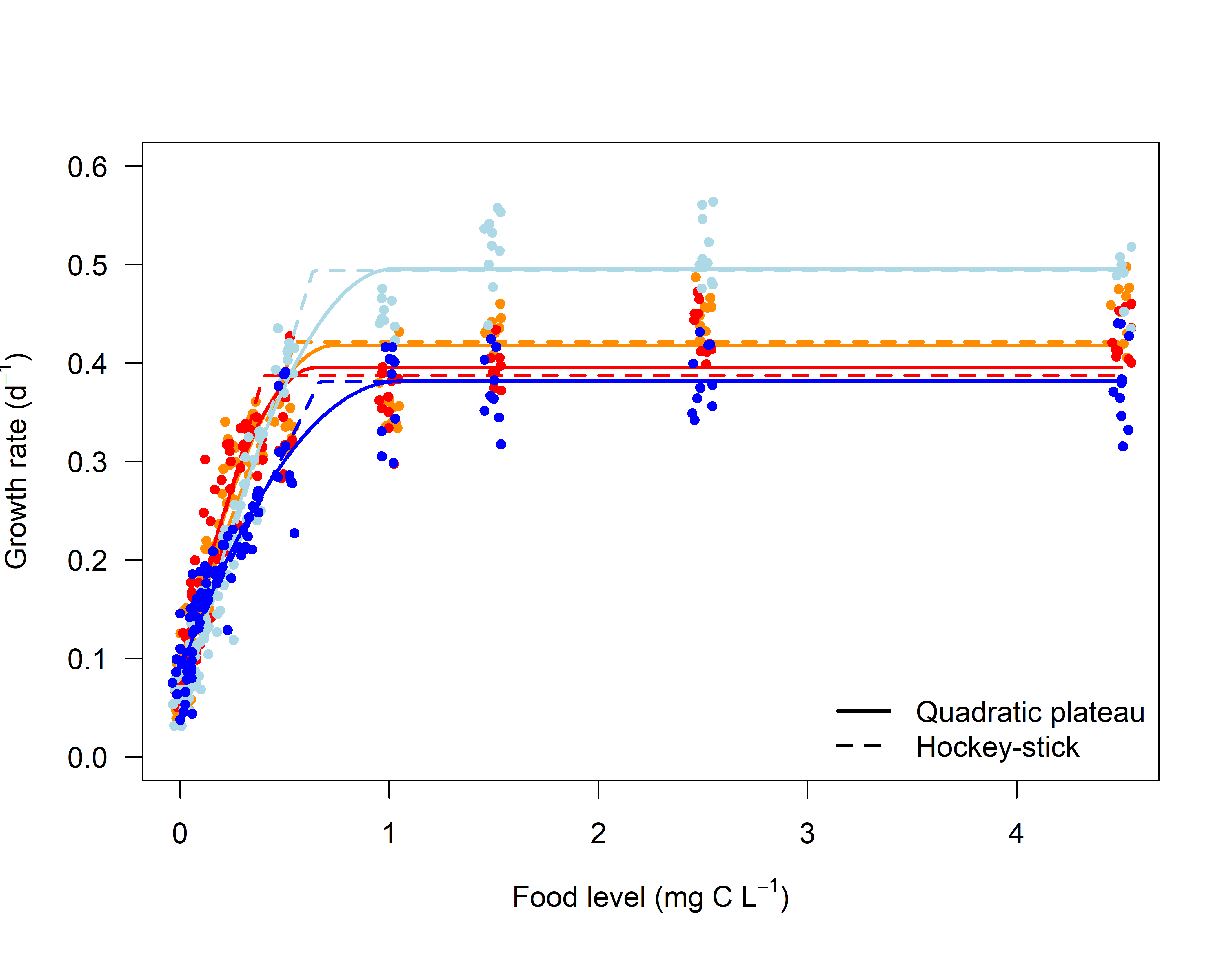

Supplement: fbag022_Supplemental_Files [file fbag022_supplemental_files.zip › FigS1_growth_QP_vs_HS_fbag022.tiff]

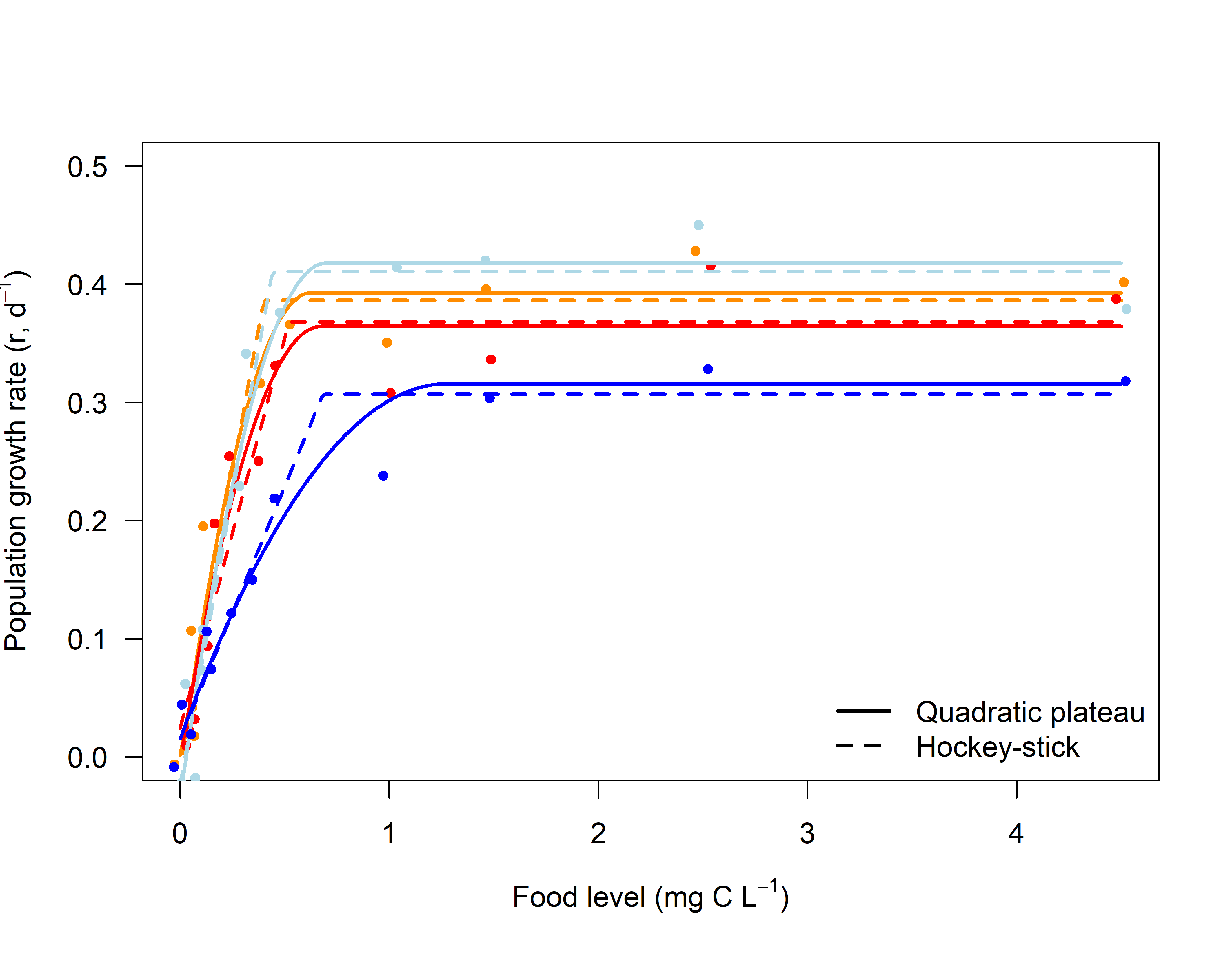

Supplement: fbag022_Supplemental_Files [file fbag022_supplemental_files.zip › FigS2_r_QP_vs_HS_fbag022.tiff]
